# Early post-bevacizumab change in rCBV from DSC-MRI identifies pseudoresponse in recurrent glioblastoma: Results from ACRIN 6677/RTOG 0625

**DOI:** 10.3389/fonc.2023.1061502

**Published:** 2023-01-26

**Authors:** Jerrold L. Boxerman, Bradley S. Snyder, Daniel P. Barboriak, Kathleen M. Schmainda

**Affiliations:** ^1^ Department of Diagnostic Imaging, Rhode Island Hospital and Alpert Medical School of Brown University, Providence, RI, United States; ^2^ Center for Statistical Sciences, Brown University School of Public Health, Providence, RI, United States; ^3^ Department of Radiology, Duke University Medical Center, Durham, NC, United States; ^4^ Department of Biophysics, Medical College of Wisconsin, Milwaukee, WI, United States; ^5^ Department of Radiology, Medical College of Wisconsin, Milwaukee, WI, United States

**Keywords:** recurrent glioblastoma, bevacizumab, pseudoresponse, DSC-MRI, (CBV) cerebral blood volume

## Abstract

**Background:**

Progressive enhancement predicted poor survival in ACRIN 6677/RTOG 0625, a multi-center trial of bevacizumab with irinotecan or temozolomide in recurrent glioblastoma, but pseudoresponse likely limited enhancement-based survival prognostication in T1 non-progressors. We aimed to determine whether early change in cerebral blood volume from baseline (ΔCBV) could further stratify the T1 non-progressors according to overall (OS) and progression-free (PFS) survival.

**Methods:**

37/123 enrolled patients had DSC-MRI, including 13, 15, and 8 patients without 2D-T1 progression at 2, 8, and 16 weeks post-treatment initiation, respectively. Mean CBV normalized to white matter (nRCBV) and mean standardized CBV (sRCBV) were extracted from enhancing tumor. ROC curves were derived for ΔCBV using six-month PFS and one-year OS as reference standards. Kaplan-Meier survival estimates and log-rank test compared PFS and OS for both ΔCBV (increase vs. decrease) and T1 response status (stable vs. decreasing enhancement).

**Results:**

PFS and OS were significantly worse for increasing CBV at 2 weeks (p=0.003 and p=0.002 for nRCBV, and p=0.03 and p=0.03 for sRCBV, respectively), but not for 2D-T1 patients with stable vs. decreasing enhancement (p=0.44 and p=0.86, respectively). ΔCBV at week 2 was also a good prognostic marker for OS-1 and PFS-6 using ROC analysis. By contrast, 2D-T1 response status at weeks 2, 8, and 16 was not associated with PFS-6. ΔCBV at 16 weeks (p=0.008 for sRCBV) but not 8 weeks (p=0.74 for nRCBV and p=0.56 for sRCBV) was associated with significant difference in median survival, but no difference in survival was observed for 2D-T1 patients with stable vs. decreasing enhancement at 8 weeks (p=0.69) or 16 weeks (p=0.21). At 16 weeks, OS did not differ significantly between 2D-T1 progressors and 2D-T1 non-progressors with increasing CBV (median survival 3.3 months post week 16 scan vs. 9.2 months, respectively; p=0.13), suggesting that 2D-T1 non-progressors with increasing CBV may have a prognosis like that of 2D-T1 progressors.

**Conclusion:**

After 2 weeks of anti-angiogenic therapy, ΔCBV in 2D-T1 non-progressors significantly prognosticated PFS and OS, whereas 2D-T1 response status did not, identifying a subpopulation that benefits from bevacizumab. Combining 2D-T1 progression and ΔCBV may yield a response assessment paradigm with 3-tiered OS stratification.

## 1 Introduction

Glioblastoma, the most common and aggressive primary brain tumor, has dismal prognosis with median overall survival (OS) of 12-15 months and 5-year survival rate of 9.8% (1). Maximal safe resection plus chemoradiation with concomitant and adjuvant temozolomide is standard of care (1), but rapid recurrence is typical (2). Glioblastoma typically overexpresses vascular endothelial growth factor (VEGF), motivating anti-angiogenic treatment trials. Bevacizumab, a humanized monoclonal anti-VEGF antibody (3), conferred progression-free survival (PFS) benefit versus historic controls (4), but clinical trials have failed to demonstrate OS benefit for newly diagnosed (5, 6) or recurrent (7) glioblastoma. Nonetheless, recent evidence suggests that bevacizumab may improve OS in a subset of patients (8–11), thus making early post-treatment imaging biomarkers that can predict response potentially important.

Imaging assessment of response to antiangiogenic therapy is challenging because VEGF inhibitors quickly decrease vascular permeability and suppress contrast enhancement (12). This “pseudoresponse” may not reflect decreased tumor burden, limiting objective response based on contrast-enhanced T1-weighted MRI as a predictor of OS (13, 14), and prompting inclusion of FLAIR in modified response criteria (15). The ACRIN 6677/RTOG 0625 central reader study demonstrated that although progressive contrast enhancement in recurrent glioblastoma after 2-4 cycles of anti-VEGF therapy prognosticated poor survival, there was no significant survival benefit for contrast enhancement responders (decreasing enhancement) compared to non-responders, non-progressors (stable disease) (16). Whereas progressive enhancement identified relative bevacizumab failures, regressive enhancement failed to sub-select the non-progressors likely to do well. Presence of progressive disease on FLAIR imaging was also unable to sub-stratify the T1 non-progressors (16).

Physiologic imaging markers such as relative cerebral blood volume (rCBV) from DSC-MRI may more accurately predict treatment response. Absolute pre- and post-treatment rCBV in single-institution studies (17–19) and change in rCBV from baseline in ACRIN 6677/RTOG 0625 (20) predicted OS in bevacizumab-treated recurrent glioblastoma regardless of T1 progression status.

In this study, we re-evaluated the ACRIN 6677/RTOG 0625 DSC-MRI studies for 2D-T1 non-progressors to determine whether early change in rCBV better predicts OS and PFS than 2D-T1 response status, specifically addressing the problem of pseudoresponse in responders versus non-responders. We also explored how change in rCBV for 2D-T1 non-progressors might be added to a response assessment paradigm based on 2D-T1 progression.

## 2 Materials and methods

The Radiation Therapy Oncology Group (RTOG, now NRG Oncology), in collaboration with the American College of Radiology Imaging Network (ACRIN, now ECOG-ACRIN), both funded by the National Cancer Institute, conducted a prospective, randomized phase II multi-center trial to evaluate bevacizumab with irinotecan or temozolomide in recurrent glioblastoma (ACRIN 6677/RTOG 0625). Twenty-three institutions participated in this HIPAA-compliant trial after obtaining IRB approval. Informed consent was obtained for all patients.

### 2.1 Study subjects

All patients had recurrent histologically proven glioblastoma or gliosarcoma (pre-WHO 2016 classification). Detailed inclusion and exclusion criteria have been published (16). Patients received bevacizumab (10 mg/kg IV, days 1 and 15 of a 28-day cycle) and were randomized to receive either temozolomide (75 mg/m^2^ p.o., days 1-21 during the first 28-day cycle; 100 mg/m^2^ for cycle 2 and beyond) or irinotecan (125 mg/m^2^ IV, days 1 and 15 of a 28-day cycle); both treatment arms were pooled for this study due to small sample size.

### 2.2 MRI protocol

Imaging was performed at 1.5T (Siemens Espree, Siemens Avanto, GE Signa Excite, GE HDx) or 3T (GE HDx, GE Excite). Conventional MRI included pre-contrast T1-weighted, T2-weighted, FLAIR, and diffusion-weighted imaging. After intravenous injection of 0.1 mmol/kg of standard gadolinium-based agent, axial 2D spin-echo (2D-T1) and 3D gradient-echo (3D-T1) T1-weighted images were acquired. Contrast agent administered for conventional post-contrast imaging served as “pre-load” for subsequent DSC-MRI, potentially diminishing contrast extravasation-associated T1 contribution to DSC-MRI signal (21–23). Echo-planar gradient-echo (TE=30-40ms) DSC-MRI was performed with TR=1.3-1.5s (120 repetitions), flip angle=90°, slice thickness=5mm (0-2.5mm gap), matrix=128x128, and FOV=22-24cm. Images were acquired for 1 min before and 2 min after bolus injection of 0.1 mmol/kg gadolinium-based contrast agent. Imaging was performed at baseline, 2 weeks after bevacizumab initiation, and after 2 and 4 treatment cycles (8 and 16 weeks). Complete MRI parameters are on the ACRIN website (https://www.acr.org/Research/Clinical-Research/ACRIN-Legacy-Trials).

### 2.3 Central reader methods

Central reader methods were previously described (16). All local imaging was retrospectively transmitted to ACRIN for central review. Two primary readers independently measured 2D-T1 largest diameter of contrast enhancement and maximum perpendicular diameter for each target lesion. Time of 2D-T1 progression, and radiologic response status at each time point were determined using Macdonald (24) and RANO (25) threshold criteria. Steroid dosage and clinical status were unavailable to ACRIN readers. An adjudicator settled discordant times to progression.

### 2.4 rCBV computation and image analysis

Normalized and standardized rCBV (nRCBV, sRCBV) maps were computed using OsiriX open-source software with the IB Neuro™ plug-in (Imaging Biometrics LLC, Elm Grove, WI). On a voxel-wise basis, baseline pre-bolus mean signal intensity was determined, omitting the five initial time points; the truncated signal-time series was converted to a relaxivity-time series, ΔR2*(*t*); rCBV was estimated using trapezoidal integration of ΔR2*(*t*) over post-bolus time points (first-pass plus post-bolus tail) and a post-processing leakage correction algorithm (21–23). rCBV maps were normalized to mean rCBV (nRCBV) in contralateral normal-appearing white matter consistently located across all longitudinal studies, and standardized rCBV (sRCBV) maps were produced using a published technique (26).

For semi-automatic lesion segmentation, we manually defined the region of lesion enhancement, excluding hemorrhage and macrovessels, on difference images computed from co-registered standardized pre- and post-contrast T1-weighted images using IB Delta Suite™ (Imaging Biometrics LLC, Elm Grove, WI). We further constrained regions of enhancement using empirical thresholds, excluding central necrosis, and edited segmentations to exclude non-lesion voxels (27). These ROIs were applied to nRCBV and sRCBV maps co-registered to the 2D-T1 images, from which mean values were extracted. This process was repeated with new ROIs at each time point.

### 2.5 Statistical methods

At weeks 2, 8 and 16, we analyzed patients who did not progress on 2D-T1. Patients were classified as decreasing enhancement if both readers rated partial or complete response by the specified time point; otherwise, patients were classified as stable enhancement. Percent change in nRCBV and sRCBV were calculated from baseline for each time point, and summary statistics including mean, standard deviation, median and range were computed.

To assess predictive ability and discrimination, receiver operating characteristic (ROC) curves were derived for continuous percent change in nRCBV and sRCBV at each time point, using OS status at 1 year (OS-1) and PFS status at 6 months (PFS-6) as reference standards, where OS-1 and PFS-6 were defined from patient registration. Area under the ROC curve (AUC) and associated 95% confidence intervals were computed empirically. As 2D-T1 response status is not a continuous marker, ROC analysis cannot be performed; instead, association with OS-1 and PFS-6 was examined using Fisher’s exact test.

To assess the association with PFS and OS generally, and to compare survival times, Kaplan-Meier estimates and the log-rank test were used to compare groups defined using percent change in nRCBV and sRCBV (increase, ≥0, vs. decrease, <0) and 2D-T1 response status (decreasing enhancement vs. stable enhancement). For the time-to-event Kaplan-Meier analysis, OS and PFS were calculated from the date of the respective scan for each of week 2, week 8 and week 16.

For week 16 data, an exploratory analysis was also conducted, using both 2D-T1 response status and percent change in nRCBV to create a three-tier patient stratification: 2D-T1 progression, 2D-T1 non-progression with increased nRCBV, and 2D-T1 non-progression with decreased nRCBV. Kaplan-Meier estimates were computed, and the log-rank test was used to compare time to death.

Statistical computations were performed using SAS 9.4 (SAS Institute, Cary, NC) or R version 4.1.3 (R project: http://www.r-project.org/). All statistical tests were two-sided, with p-values <0.05 considered statistically significant. As the reported analyses are *post hoc* and were considered hypothesis-generating, no adjustment was made for multiplicity of inference.

## 3 Results

### 3.1 Study cohort

The entire ACRIN 6677 study cohort with DSC-MRI results was described previously (16). A total of 37/123 enrolled patients had DSC-MRI, 21 of whom had data sufficient for analysis, defined as having both a baseline and at least one post-baseline scan with interpretable DSC-MRI. After excluding 2D-T1 progressors, there were 13, 15, and 8 2D-T1 non-progressors available for analysis at weeks 2, 8, and 16, respectively. There were no 2D-T1 progressors at week 2. **Table 1** provides salient demographic and clinical data for analyzed patients, including age, sex, surgery, location of primary tumor, treatment arm, and indicators for availability of week 2, week 8 and week 16 DSC-MRI data. **Table 2** provides summary statistics of the percent change in nRCBV and sRCBV at each time point.

**Table 1:** Demographic and clinical data for the cohort of 2D-T1 non-progressors with available week 2, week 8 or week 16 DSC-MRI data.

Subject	Age	Sex	KPS	Type of Surgery (Initial GBM)	Additional Surgery (Recurrent GBM)	Location of Primary Tumor								Treatment arm	Week 2 DSC-MRI (n=13)	Week 8 DSC-MRI (n=15)	Week 16 DSC-MRI (n=8)
						Frontal	Temporal	Parietal	Occipital	Basal Ganglia	Cerebellum	Brainstem	Corpus Callosum				
**1**	55	Male	90	Subtotal resection	None		X							Bev+CPT-11	0	1	0
**2**	45	Male	100	Total Tumor Resection	None		X	X						Bev+CPT-11	0	1	0
**3**	51	Female	80	Total Tumor Resection	Total Tumor Resection		X							Bev+CPT-11	0	1	1
**4**	48	Female	70	Subtotal resection	None	X								Bev+TMZ	0	1	1
**5**	36	Male	100	Total Tumor Resection	None		X	X	X					Bev+CPT-11	0	1	1
**6**	62	Male	70	Missing	None	X								Bev+CPT-11	0	1	1
**7**	58	Male	100	Total Tumor Resection	Total Tumor Resection			X						Bev+TMZ	1	0	0
**8**	39	Male	80	Total Tumor Resection	None		X	X		X		X	X	Bev+CPT-11	1	0	0
**9**	74	Female	100	Biopsy only	None		X	X	X		X			Bev+CPT-11	1	0	0
**10**	68	Female	80	Total Tumor Resection	None		X							Bev+TMZ	1	0	0
**11**	64	Male	90	Total Tumor Resection	None		X	X	X				X	Bev+TMZ	1	1	0
**12**	60	Female	80	Subtotal resection	Subtotal Resection		X	X						Bev+CPT-11	1	1	0
**13**	43	Female	70	Total Tumor Resection	None	X		X					X	Bev+CPT-11	1	1	0
**14**	60	Male	90	Total Tumor Resection	Total Tumor Resection		X		X					Bev+TMZ	1	1	0
**15**	62	Female	100	Subtotal resection	None		X	X						Bev+TMZ	1	1	0
**16**	23	Male	70	Subtotal resection	None	X								Bev+CPT-11	1	1	1
**17**	58	Female	80	Total Tumor Resection	Total Tumor Resection	X								Bev+CPT-11	1	1	1
**18**	43	Male	100	Total Tumor Resection	None			X						Bev+TMZ	1	1	1
**19**	51	Male	70	Biopsy only	None	X								Bev+TMZ	1	1	1

KPS, Karnofsky Performance Status; GBM, Glioblastoma Multiforme; DSC-MRI, Dynamic Susceptibility Contrast MR Perfusion; Bev, Bevacizumb; TMZ, Temozolomide; CPT-11, Irinotecan.

The "X" designates which of the 8 identified brain regions were involved with tumor for the particular case.

**Table 2:** Summary statistics of percent change in nRCBV and sRCBV by time point for the cohort of 2D-T1 non-progressors, overall and stratified by patient outcome (OS-1 and PFS-6).

Week	Parameter		N	nRCBV		sRCBV	
				Mean (SD)	Median [Range]	Mean (SD)	Median [Range]
2	All patients		13	-17.1 (54.5)	-11.6 [-89.8, 81.8]	-16.1 (54.1)	-37.4 [-91.5, 86.8]
	OS-1	Alive	5	-52.9 (33.5)	-64.7 [-89.8, -11.6]	-56.4 (21.6)	-51.7 [-91.5, -37.4]
		Dead	8	5.2 (54.4)	0.3 [-78.5, 81.8]	9.2 (53.5)	9.1 [-61.0, 86.8]
	PFS-6	No Progression	6	-57.2 (31.7)	-69.5 [-89.8, -11.6]	-57.2 (19.4)	-56.0 [-91.5, -37.4]
		Progression ^1^	6	12.7 (48.6)	0.3 [-53.6, 81.8]	7.9 (42.6)	9.1 [-54.2, 61.0]
8	All patients		15	-20.6 (49.8)	-40.5 [-96.1, 68.8]	-24.7 (36.8)	-19.1 [-93.4, 42.7]
	OS-1	Alive	6	-13.9 (53.8)	-38.9 [-65.2, 68.8]	-25.2 (23.5)	-23.5 [-51.3, 8.2]
		Dead	9	-25.0 (49.8)	-46.8 [-96.1, 55.8]	-24.3 (45.0)	-19.1 [-93.4, 42.7]
	PFS-6	No Progression	10	-32.9 (48.9)	-46.8 [-96.1, 68.8]	-31.3 (35.5)	-41.0 [-93.4, 30.5]
		Progression ^2^	4	21.6 (29.2)	16.8 [-3.0, 55.8]	2.7 (27.9)	-6.3 [-19.1, 42.7]
16	All patients		8	-2.2 (44.7)	-11.5 [-53.6, 56.7]	-13.7 (27.3)	-14.0 [-60.9, 33.8]
	OS-1	Alive	6	-12.9 (44.3)	-31.9 [-53.6, 54.7]	-22.6 (22.1)	-20.6 [-60.9, -0.003]
		Dead	2	29.8 (38.0)	29.8 [3.0, 56.7]	13.2 (29.2)	13.2 [-7.5, 33.8]
	PFS-6	No Progression	7	-10.6 (40.9)	-26.0 [-53.6, 54.7]	-20.5 (20.9)	-20.5 [-60.9, -0.003]
		Progression ^3^	0	N/A	N/A	N/A	N/A

^1^ Excludes one patient who progressed clinically prior to week 2.

^2^ Excludes one patient deemed by local site to have progressed based on the week 8 scan.

^3^ Excludes one patient deemed by local site to have progressed based on the week 16 scan. After this patient was excluded there were no remaining patients with progression.

nRCBV, normalized relative cerebral blood volume; sRCBV, standardized relative cerebral blood volume; OS-1, overall survival at 1 year; PFS-6, progression-free survival at 6 months; SD, standard deviation.

N/A, Not applicable.

### 3.2 Prediction of progression-free survival

**Figure 1:**
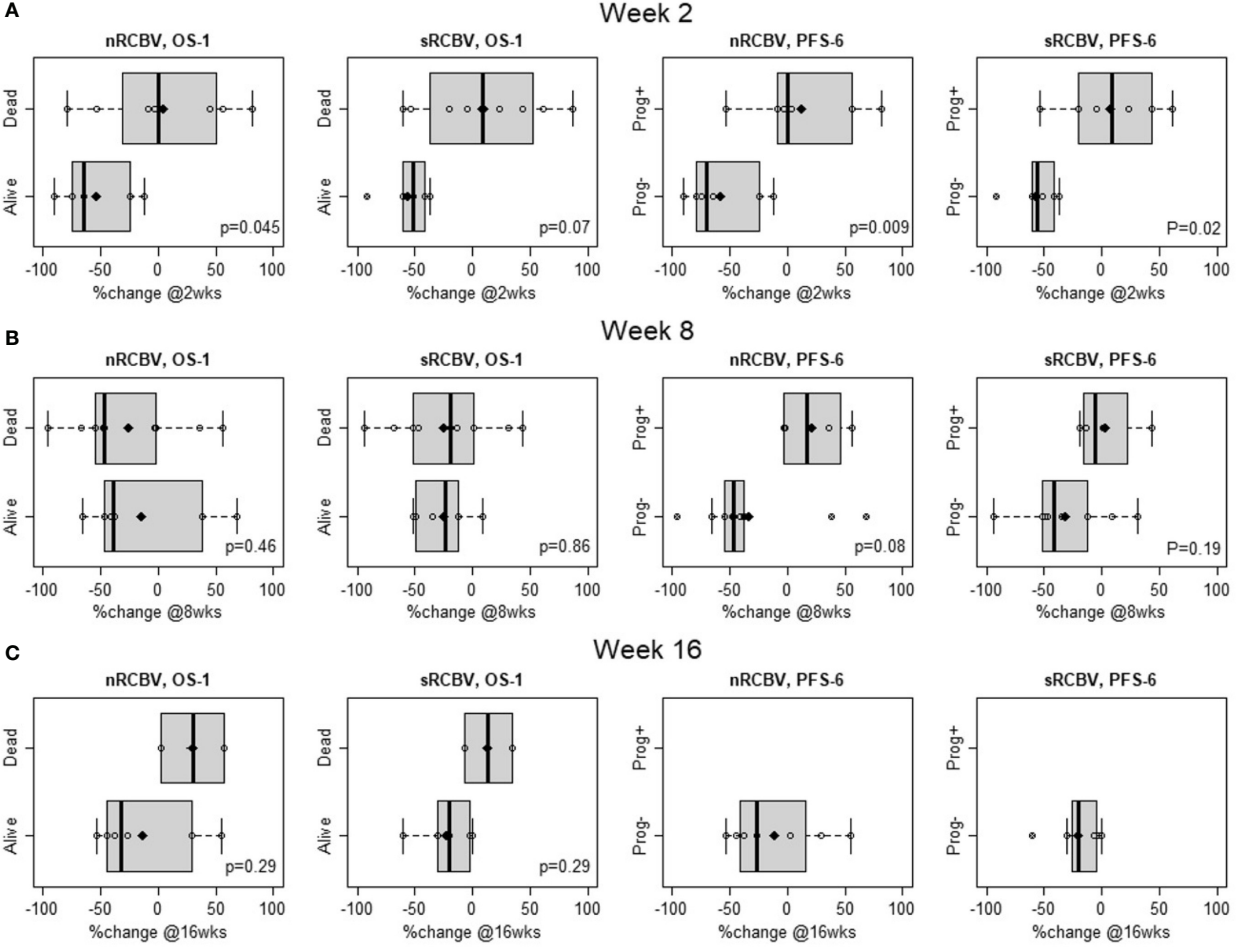
Distribution of percent change in normalized rCBV (nRCBV) and standardized rCBV (sRCBV) from baseline to week 2 **(A)**, week 8 **(B)**, and week 16 **(C)** for 2D-T1 non-progressors by overall survival status at 1 year (OS-1) and progression-free survival status at 6 months (PFS-6).

We evaluated the ability of DSC MRI to predict PFS among 2D-T1 non-progressors at weeks 2, 8 and 16 separately using two different approaches. First, we determined whether percent change in nRCBV and sRCBV predict PFS-6 using ROC analysis. There were 12 and 14 patients at week 2 and 8, respectively, whose progression status was confirmed after the respective scan and before 6 months. Of those patients, 6/12 (50%) and 10/14 (71%) were progression-free at 6 months; none of the 2D-T1 non-progressors at week 16 had progressed at 6 months, precluding ROC analysis (**Table 2**). **Figure 1** shows the distribution of percent change in nRCBV and sRCBV at week 2 (A), week 8 (B), and week 16 (C) by PFS-6 status. **Table 3** provides estimates of ROC AUC using PFS-6 as the reference standard, along with associated 95% confidence intervals. Percent change in nRCBV and sRCBV at week 2 exhibited high AUC values (AUC [95% CI] = 0.94 [0.82–1] and 0.92 [0.74–1], respectively), indicating very good discrimination. Corresponding values at week 8 were lower (AUC [95% CI] = 0.83 [0.59–1] and 0.75 [0.47–1], respectively). By contrast, 2D-T1 response status (stable vs. decreasing enhancement) at weeks 2 and 8 was not associated with PFS-6 in the subset of 2D-T1 non-progressors (p=1.0 and p=0.58, respectively; cross tabulations not shown).

**Table 3:** Empirical ROC AUC for percent change in nRCBV and sRCBV by time point and patient outcome (OS-1 and PFS-6) among the cohort of 2D-T1 non-progressors.

Outcome	Week	N	ROC AUC [95% CI]	
			nRCBV	sRCBV
OS-1	2	13	0.85 [0.62–1]	0.83 [0.58–1]
	8	15	0.63 [0.32–0.94]	0.54 [0.23–0.85]
	16	8	0.83 [0.45–1]	0.83 [0.45–1]
PFS-6	2	12	0.94 [0.82–1]	0.92 [0.74–1]
	8	14	0.83 [0.59–1]	0.75 [0.47–1]
	16	7	N/A ^1^	N/A ^1^

^1^None of the patients with week 16 imaging had progressed at 6 months, thus precluding ROC analysis.

nRCBV, normalized relative cerebral blood volume; sRCBV, standardized relative cerebral blood volume; ROC AUC, area under the receiver operating characteristic curve; OS-1, overall survival at 1 year; PFS-6, progression-free survival at 6 months.

N/A, Not applicable.

**Figure 2:**
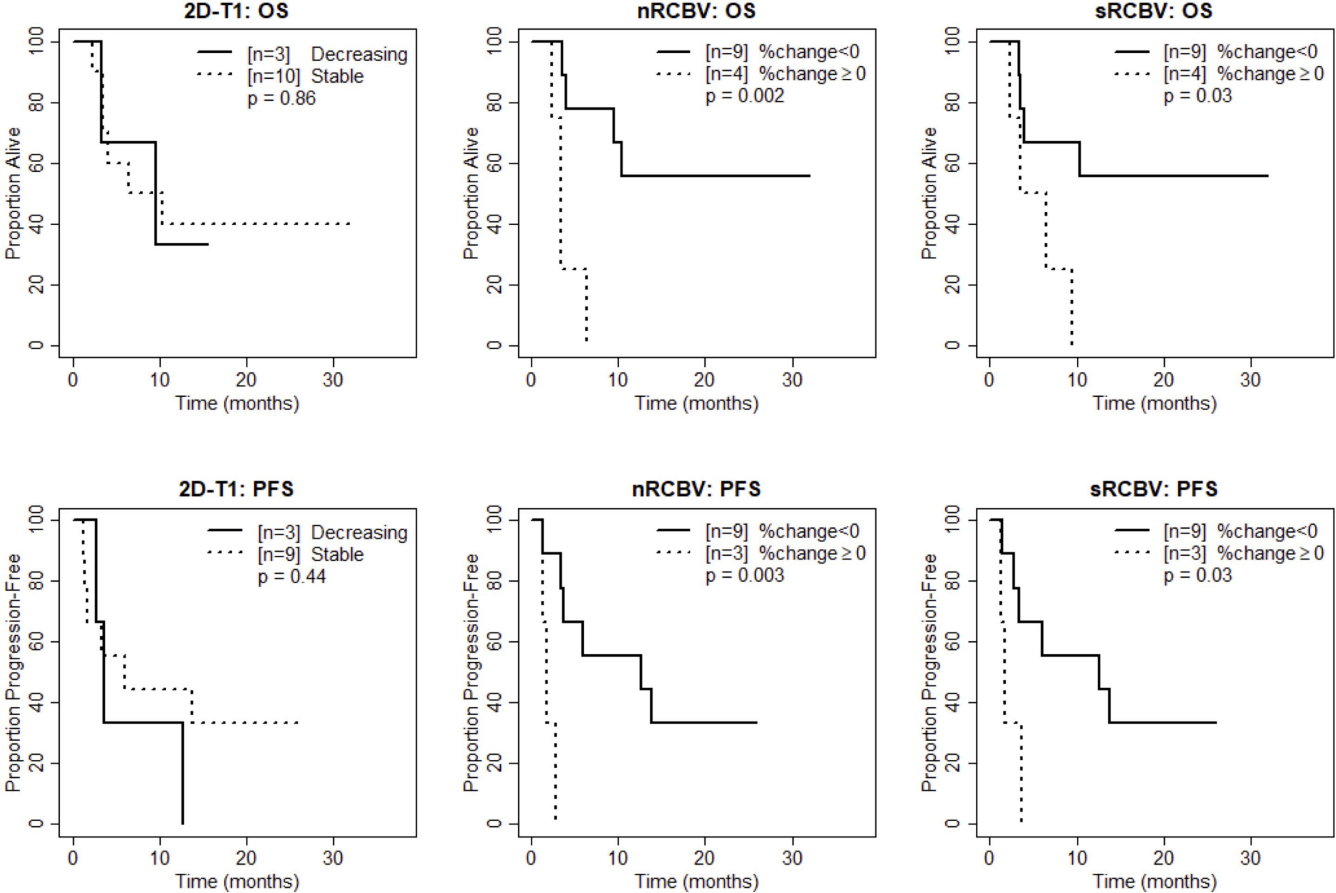
Kaplan-Meier curves using week 2 DSC-MRI for time to death (top row) and time to progression or death (bottom row) for T1 non-progressors with stable versus decreasing enhancement on 2D-T1 (left column), T1 non-progressors with decreasing versus increasing normalized rCBV (nRCBV) (middle column), and T1 non-progressors with decreasing versus increasing standardized rCBV (sRCBV) (right column).

**Figure 3:**
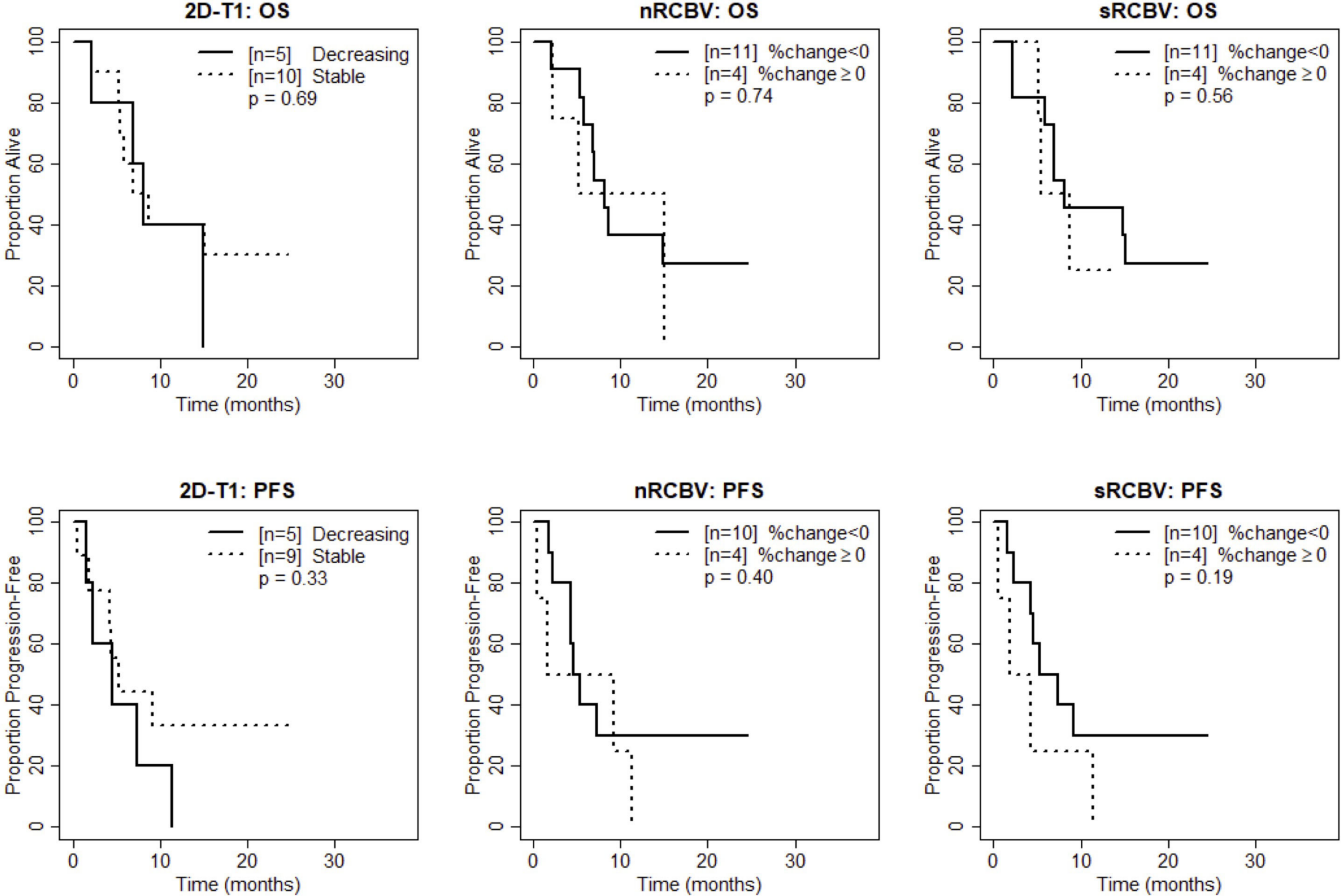
Kaplan-Meier curves using week 8 DSC-MRI for time to death (top row) and time to progression or death (bottom row) for T1 non-progressors with stable versus decreasing enhancement on 2D-T1 (left column), T1 non-progressors with decreasing versus increasing normalized rCBV (nRCBV) (middle column), and T1 non-progressors with decreasing versus increasing standardized rCBV (sRCBV) (right column).

**Figure 4:**
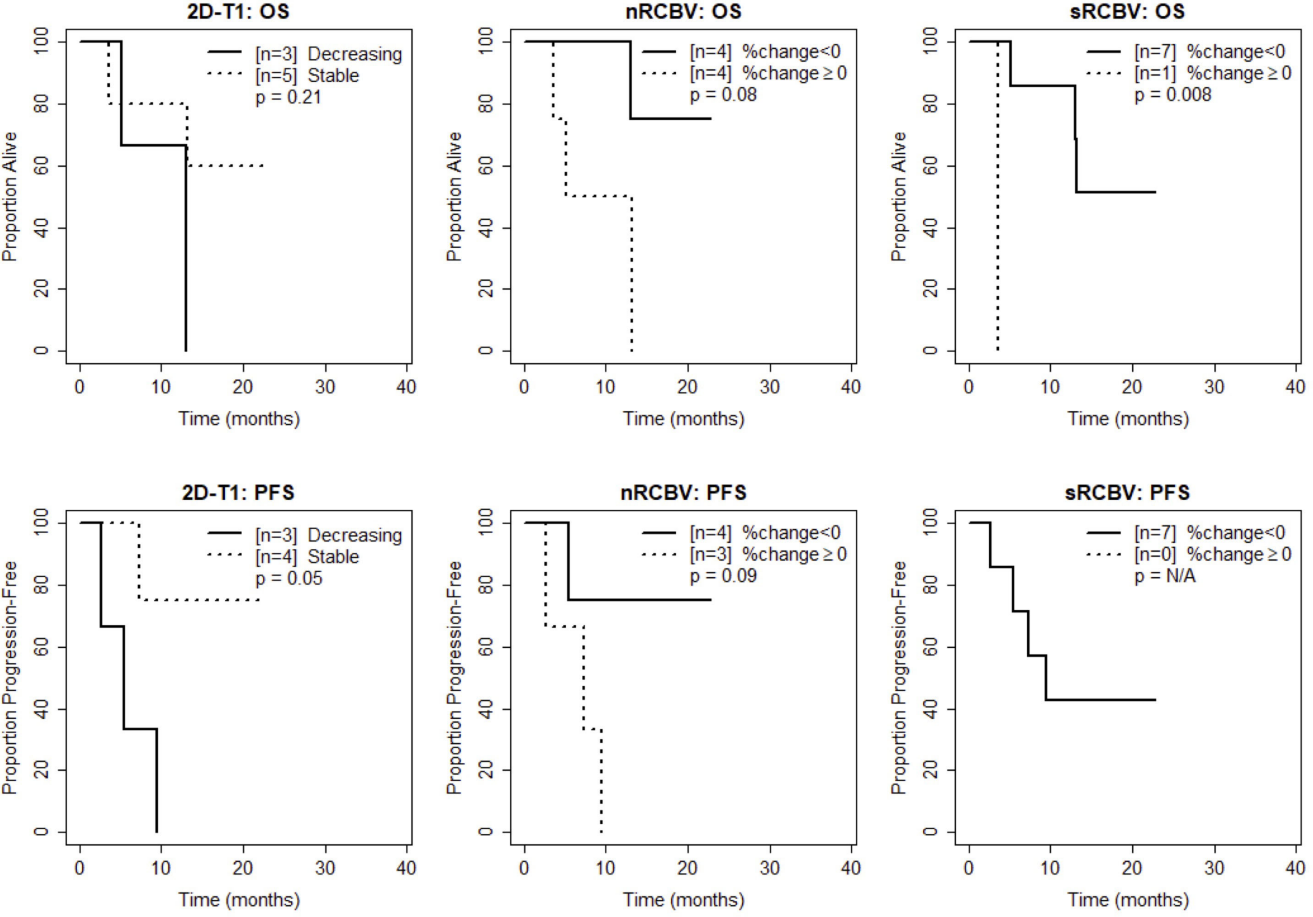
Kaplan-Meier curves using week 16 DSC-MRI for time to death (top row) and time to progression or death (bottom row) for T1 non-progressors with stable versus decreasing enhancement on 2D-T1 (left column), T1 non-progressors with decreasing versus increasing normalized rCBV (nRCBV) (middle column), and T1 non-progressors with decreasing versus increasing standardized rCBV (sRCBV) (right column).

We also compared time to progression/death after dichotomizing patients with increase (≥0) vs. decrease (<0) in nRCBV and sRCBV. At week 2, patients with increased blood volume had significantly shorter PFS compared to patients with decreased blood volume for both nRCBV (median 1.7 months post week 2 scan vs. 12.6 months, p=0.003) and sRCBV (median 1.7 months post week 2 scan vs. 12.6 months, p=0.03) (**Figure 2**). By comparison, PFS did not differ significantly between groups based on 2D-T1 response status (median 3.7 months post week 2 scan for decreasing enhancement vs. 6.4 months for stable enhancement, respectively, p=0.44) (**Figure 2**). At week 8, neither percent change in nRCBV (p=0.40), percent change in sRCBV (p=0.19), nor 2D-T1 response status (p=0.33) were associated with PFS (**Figure 3**). At week 16, there were no patients with increase in sRCBV which precluded comparison of PFS. nRCBV was not predictive of PFS at week 16 (p=0.09), but 2D-T1 response status was borderline significant (p= 0.05) (**Figure 4**); however, PFS did not differ by 2D-T1 response status among the entire cohort of 2D-T1 non-progressors at week 16 (p=0.66, n=45), of which this small number of patients also with DSC-MRI data (n=7) was a subset.

These results suggest that at week 2, among 2D-T1 non-progressors, percent change in nRCBV and sRCBV are good prognostic markers for both PFS-6 (evaluated as continuous markers) and PFS in general (dichotomized as increase vs. decrease in rCBV) and outperform the 2D-T1 response criteria. At week 8, neither nRCBV nor sRCBV could further distinguish PFS in general (dichotomized as increase vs. decrease in rCBV), and the 2D-T1 response criteria are not useful. At week 16, nRCBV did not yield a statistically significant difference in PFS (dichotomized as increase vs. decrease in rCBV), although the sample size at this time point was small.

### 3.3 Prediction of overall survival

The ability of percent change in nRCBV and sRCBV to predict OS at week 2 and week 16 in the entire patient cohort was previously described (20); here, we test whether it can predict OS in the subset of 2D-T1 non-progressors. Because no patients progressed at week 2, our week 2 cohort is identical to that published previously (20), where only OS data were presented. Schmainda et al. showed that percent change in nRCBV at week 2 differed significantly between patients who died by 1 year versus those who did not (p=0.045, ROC AUC [95% CI] = 0.85 [0.62–1]), and that patients with increased nRCBV at week 2 had poorer OS compared to those with decreased nRCBV (p=0.002), with a similar finding at week 2 for sRCBV (p=0.03). For clarity and ease of presentation, these results are re-incorporated into the findings for the subset of 2D-T1 non-progressors below.

**Figure 1** shows the distribution of percent change in nRCBV and sRCBV at week 2 (A), week 8 (B), and week 16 (C) by OS-1 status. Estimates of ROC AUC using OS-1 as the reference standard, along with associated 95% CIs, are shown in **Table 3**. Percent change in nRCBV (AUC [95% CI] = 0.85 [0.62–1]) and sRCBV (AUC [95% CI] = 0.83 [0.58–1]) at week 2 are good prognostic markers of OS-1. AUC estimates at week 16 were similar but exhibited wider confidence intervals due to the smaller number of available patients; AUC estimates at week 8 were poor. Response status as determined by 2D-T1 at weeks 2, 8 and 16 were not associated with OS-1 (p=1.0, p=1.0, and p=1.0, respectively; cross tabulations not shown).

At week 2, patients with increased blood volume had significantly shorter survival compared to patients with decreased blood volume for both nRCBV (median 3.4 months post week 2 scan vs. not reached, p=0.002) and sRCBV (median 5.0 months post week 2 scan vs. not reached, p=0.03), whereas OS did not differ significantly based on 2D-T1 response status (p=0.86) (**Figure 2**). At week 8, neither percent change in nRCBV (p=0.74), percent change in sRCBV (n=0.56), nor 2D-T1 response status (p=0.69) were associated with OS (**Figure 3**). At week 16, patients with increased sRCBV had significantly shorter survival compared to patients with decreased sRCBV (median 3.6 months post week 16 scan vs. not reached, p=0.008), although the same comparison did not reach statistical significance for nRCBV (p=0.08) (**Figure 4**). OS did not differ significantly at week 16 based on 2D-T1 response status (p=0.21) (**Figure 4**).

These results suggest that at week 2, among 2D-T1 non-progressors, percent change in nRCBV and sRCBV are good prognostic markers for both OS-1 (evaluated as continuous markers) and OS in general (dichotomized as increase vs. decrease in rCBV), and again outperform the 2D-T1 response criteria. At week 8, neither percent change in nRCBV or sRCBV nor the 2D-T1 response criteria were predictive of survival. At week 16, percent change in sRCBV is associated with OS, although patient counts are small; 2D-T1 response status at week 16 was not predictive of survival.

### 3.4 Prediction of OS using combined 2D-T1 progression status and nRCBV

**Figure 5:**
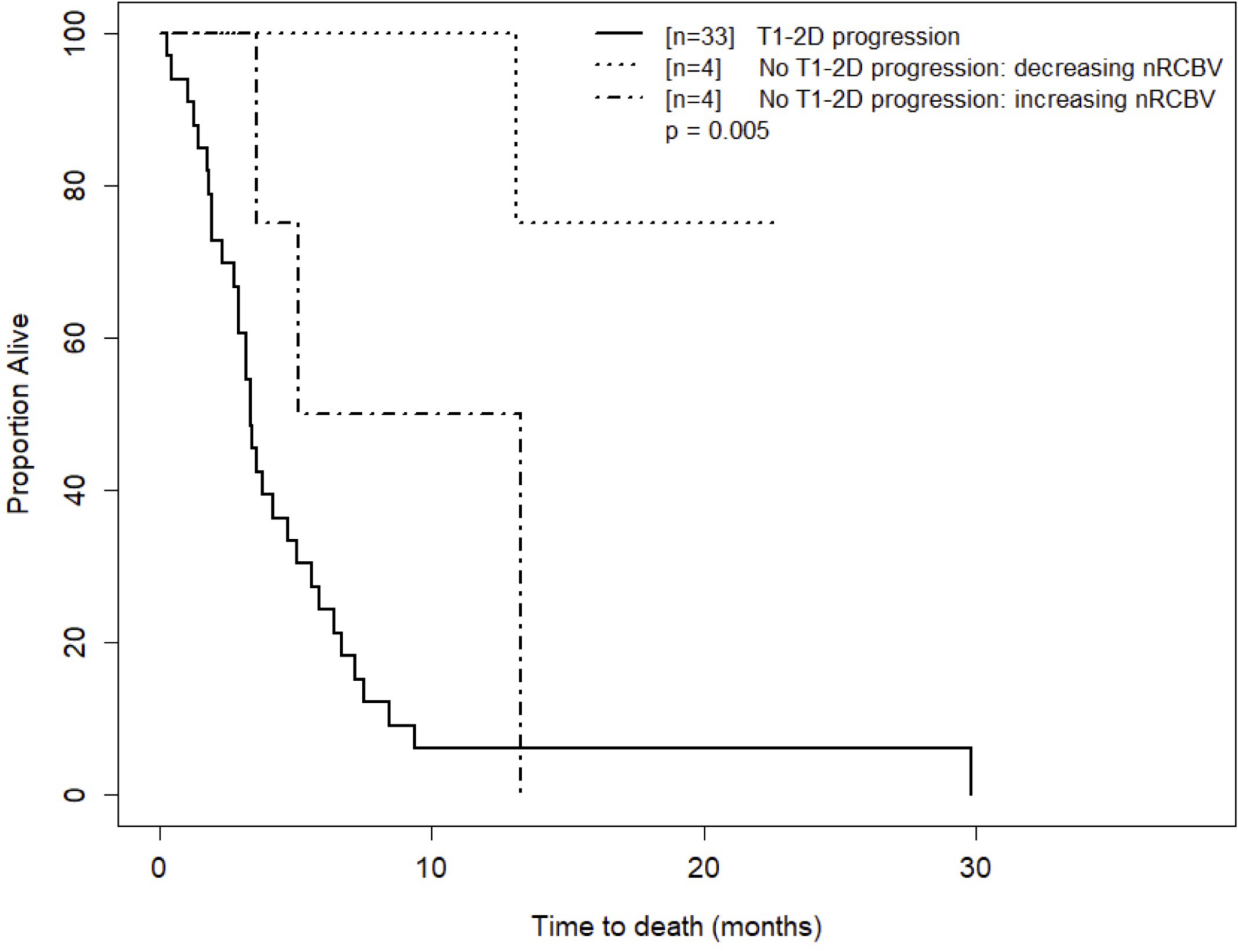
Kaplan-Meier curves for time to death for T1 progressors and T1 non-progressors with increasing versus decreasing nRCBV at week 16. Visual separation of the curves potentiates 3-tier survival stratification, and an interpretation paradigm for recurrent GBM shortly after Bevacizumab whereby patients with progressive enhancement are deemed treatment failures, and change in rCBV further distinguishes relative successes from failures.

As an exploratory analysis, we further analyzed the week 16 data to compare OS using both 2D-T1 response status and percent change in nRCBV to create a three-tier patient stratification: progression by 2D-T1 forms one patient group, and percent change in nRCBV is used to further divide the 2D-T1 non-progressors. As there were no 2D-T1 progressors at week 2 among patients with available DSC-MRI data, week 16 was selected for this analysis due to the larger number of 2D-T1 progressors (n=33), and a modest number of non-progressors with available nRCBV data (n=8, 4 with positive change from baseline). The survival curves for the stratification are shown in **Figure 5**. Overall, a statistically significant difference in OS was observed between the three defined groups (p=0.005). OS did not differ significantly between 2D-T1 progressors and 2D-T1 non-progressors with positive change in nRCBV (median survival 3.3 months post week 16 scan vs. 9.2 months, respectively; p=0.13), suggesting that 2D-T1 non-progressors with increasing nRCBV may have a prognosis like that of 2D-T1 progressors. However, this result would require validation with a larger sample size.

## 4 Discussion

To our knowledge this is the first study that looks at the utility of CBV in T1 non-progressors, specifically addressing the issue of pseudoresponse in recurrent glioblastoma treated with antiangiogenic therapy. Our results suggest that CBV can be used to distinguish outcomes in T1 non-progressors when measured early (2 weeks) and later (16 weeks) after treatment initiation with bevacizumab. The inability to show a difference at 8 weeks is consistent with another single center study (17) and requires further exploration of the underlying pathophysiology at this post-treatment time point (20). Our results identify a potential benefit of bevacizumab in a subset of patients. This is consistent with the consensus amongst neuro-oncology practitioners that bevacizumab plays an important role in the treatment of glioblastoma (28), supported by key evidence from several clinical studies.

Although the utility of bevacizumab in glioblastoma is controversial, there is some evidence of efficacy in small subgroups. For example, in a multicenter retrospective study of 814 patients who received bevacizumab for first or second recurrence of glioblastoma initially treated with standard therapy, one patient out of twelve could be classified as a long responder (median OS of 31.1 months from the start of bevacizumab) (10).

In a study of 168 primary glioblastoma patients receiving standard therapy followed by bevacizumab and/or CCNU at first recurrence, treatment with bevacizumab was associated with improved survival in patients with large tumor 2D-T1 measurements: median OS for patients treated with bevacizumab without and with CCNU was 6.71 (n=27) and 6.97 (n=36) months, respectively, versus 4.03 months (n=10) with CCNU alone. Survival advantage from bevacizumab treatment was observed only among patients with large tumor burden (29).

In a study of the effect of bevacizumab on survival of glioblastoma patients ≥ 66 years using the Survival, Epidemiology, and End Results (SEER)-Medicare database, bevacizumab exposure was associated with a lower risk of death, providing evidence that there might be a potential benefit in elderly patients with glioblastoma that appeared independent of the number of temozolomide cycles or frontline treatment with radiotherapy and temozolomide (9).

In a study of 962 bevacizumab-treated glioblastoma patients, 28 (2.9%) long-term survivors (post-bevacizumab initiation OS ≥3 years) were identified, suggesting that a small portion of glioblastoma patients can achieve long-term survival on bevacizumab therapy (30).

Despite the potential benefit of bevacizumab for a subset of patients, clinical trials have consistently shown that while bevacizumab confers a PFS benefit, it fails to demonstrate an OS benefit for newly diagnosed (5, 6) or recurrent (7) glioblastoma. These results, reported en masse and using standard imaging only, have failed to identify a subset of patients that may benefit. This is likely due to use of standard imaging that is not able to distinguish true response from pseudoresponse. The phenomenon of pseudoresponse observed with anti-angiogenic agents like bevacizumab relates to the reduction of contrast agent extravasation and discernible tumor enhancement independent of cytotoxic or cytostatic effect. By comparison, CBV measurements are independent of enhancement status, and several single institution studies using DSC-MRI have identified patients that do benefit from bevacizumab.

We explored a three-tier patient stratification that demonstrates how both standard imaging and CBV may be used in a stepwise fashion to assess treatment response. Progression by 2D-T1 forms one patient group, and percent change in nRCBV is used to further dichotomize the 2D-T1 non-progressors. With this approach, subsets of responders can be identified early after treatment initiation. Specifically, we know from the ACRIN 6677 central reader study that 2D-T1 progression while on bevacizumab is associated with poor OS (16). Applying nRCBV analysis in the non-progressors yields two additional survival stratifications (3-tier survival). Though there was a statistically significant difference in OS between the three defined groups, OS did not differ significantly between 2D-T1 progressors and 2D-T1 non-progressors with positive change in nRCBV. This suggests that 2D-T1 non-progressors with increasing nRCBV may have a prognosis more similar to that of 2D-T1 progressors than to that of the 2D-T1 non-progressors with decreasing nRCBV, further emphasizing the utility of perfusion MRI as an adjunct to conventional contrast enhanced imaging. While the analysis was done for the 16-week data because we had enough 2D-T1 progressors and non-progressors, a similar analysis could not be performed at 2 weeks since there were no 2D-T1 progressors at this earlier time point. We presume that a similar relationship would hold at 2 weeks, but this would have to be formally tested.

A limitation of the current analysis is the small sample size, particularly at week 16. This is primarily due to the limited number of patients who agreed to the optional DSC-MRI component of the ACRIN 6677/RTOG 0625 parent trial. Restricting these patients to those without progression by 2D-T1 and with evaluable DSC-MRI at both baseline and the time point in question further reduced the available number of patients. However, despite the limited number of patients, our analyses detected a signal for DSC-MRI among 2D-T1 non-progressors. It should also be noted that presented analyses were *post hoc* and were not pre-specified in the ACRIN 6677-RTOG 0625 protocol. Thus, significant findings will require validation in a larger study.

In conclusion, for GBM patients treated with bevacizumab, early post-treatment imaging biomarkers which can predict response and afford opportunity to select alternative therapies are potentially important. Measures of rCBV using DSC-MRI provide information complementary to standard imaging and seem particularly relevant for this purpose in the context of anti-angiogenic treatments with the corresponding potential of pseudoresponse.

## Data availability statement

The datasets presented in this article may be requested through ECOG-ACRIN.

## Ethics statement

Protocol ACRIN 6677/RTOG 0625 involved human participants, and was reviewed and approved by the respective institutional review board at all twenty-three participating institutions. Written informed consent was obtained for all participants.

## Author contributions

All of the authors contributed to designing and conduct of study, analysis of results and preparation of the manuscript. All authors contributed to the article and approved the submitted version.

## References

[1] Stupp R Mason WP van den Bent MJ Weller M Fisher B Taphoorn MJB. Radiotherapy plus concomitant and adjuvant temozolomide for glioblastoma. N Engl J Med (2005) 352:987–96. doi: 10.1056/NEJMoa043330 15758009

[2] Ballman KV Buckner JC Brown PD Giannini C Flynn PJ LaPlant BR. The relationship between six-month progression-free survival and 12-month overall survival end points for phase II trials in patients with glioblastoma multiforme. Neuro-Oncology (2007) 9:29–38. doi: 10.1215/15228517-2006-025 PMC1828103 17108063

[3] Duda DG Batchelor TT Willett CG Jain RK. VEGF-targeted cancer therapy strategies: current progress, hurdles and future prospects. Trends Mol Med (2007) 13:223–30. doi: 10.1016/j.molmed.2007.04.001 PMC2686126 17462954

[4] Friedman HS Prados MD Wen PY Mikkelsen T Schiff D Abrey LE. Bevacizumab alone and in combination with irinotecan in recurrent glioblastoma. J Clin Oncol (2009) 27:4733–40. doi: 10.1200/JCO.2008.19.8721 19720927

[5] Gilbert MR Sulman EP Mehta MP. Bevacizumab for newly diagnosed glioblastoma. N Engl J Med (2014) 370:2048–9. doi: 10.1056/NEJMc1403303 24849088

[6] Chinot OL de la Motte Rouge T Moore N Zeaiter A Das A Phillips H. AVAglio: Phase 3 trial of bevacizumab plus temozolomide and radiotherapy in newly diagnosed glioblastoma multiforme. Adv Ther (2011) 28:334–40. doi: 10.1007/s12325-011-0007-3 21432029

[7] Wick W Stupp R Gorlia T Bendszus M Sahm F Bromberg JE. Phase II part of EORTC study 26101: The sequence of bevacizumab and lomustine in patients with first recurrence of a glioblastoma. J Clin Oncol (2016) 34. doi: 10.1200/jco.2016.34.15_suppl.2019

[8] Liu TT Achrol AS Mitchell LA Rodriguez SA Feroze A Iv M. Magnetic resonance perfusion image features uncover an angiogenic subgroup of glioblastoma patients with poor survival and better response to antiangiogenic treatment. Neuro Oncol (2017) 19:997–1007. doi: 10.1093/neuonc/now270 28007759 PMC5570189

[9] Davies J Reyes-Rivera I Pattipaka T Skirboll S Ugiliweneza B Woo S. Survival in elderly glioblastoma patients treated with bevacizumab-based regimens in the united states. Neuro-Oncology Pract (2018) 5:251–61. doi: 10.1093/nop/npy001 PMC6655482 31385957

[10] Morisse MC Etienne-Selloum N Bello-Roufai D Blonski M Taillandier L Lorgis V. Long-term survival in patients with recurrent glioblastoma treated with bevacizumab: a multicentric retrospective study. J Neuro-Oncology (2019) 144:419–26. doi: 10.1007/s11060-019-03245-5 31325146

[11] Schmainda KM Prah MA Marques H Kim E Barboriak DP Boxerman JL. Value of dynamic contrast perfusion MRI to predict early response to bevacizumab in newly diagnosed glioblastoma: results from ACRIN 6686 multicenter trial. Neuro-Oncology (2021) 23:314–23. doi: 10.1093/neuonc/noaa167 PMC7906067 32678438

[12] Hygino da Cruz LC Rodriguez I Domingues RC Gasparetto EL Sorensen AG. Pseudoprogression and pseudoresponse: imaging challenges in the assessment of posttreatment glioma. AJNR Am J Neuroradiol (2011) 32:1978–85. doi: 10.3174/ajnr.A2397 PMC7964401 21393407

[13] Norden AD Young GS Setayesh K Muzikansky A Klufas R Ross GL. Bevacizumab for recurrent malignant gliomas: Efficacy, toxicity, and patterns of recurrence. Neurology (2008) 70:779–87. doi: 10.1212/01.wnl.0000304121.57857.38 18316689

[14] Zuniga RM Torcuator R Jain R Anderson J Doyle T Ellika S. Efficacy, safety and patterns of response and recurrence in patients with recurrent high-grade gliomas treated with bevacizumab plus irinotecan. J Neurooncol (2009) 91:329–36. doi: 10.1007/s11060-008-9718-y 18953493

[15] Wen PY Norden AD Drappatz J Quant E. Response assessment challenges in clinical trials of gliomas. Curr Oncol Rep (2010) 12:68–75. doi: 10.1007/s11912-009-0078-3 20425610

[16] Boxerman JL Zhang Z Safriel Y Larvie M Snyder BS Jain R. Early post-bevacizumab progression on contrast-enhanced MRI as a prognostic marker for overall survival in recurrent glioblastoma: Results from the ACRIN 6677/RTOG 0625 central reader study. Neuro-Oncology (2013) 15:945–54. doi: 10.1093/neuonc/not049 PMC3688018 23788270

[17] Schmainda KM Prah M Connelly J Rand SD Hoffman RG Mueller W. Dynamic-susceptibility contrast agent MRI measures of relative cerebral blood volume predict response to bevacizumab in recurrent high-grade glioma. Neuro-Oncology (2014) 16:880–8. doi: 10.1093/neuonc/not216 PMC4022214 24431219

[18] Kickingereder P Wiestler B Burth S Wick A Nowosielski M Heiland S. Relative cerebral blood volume is a potential predictive imaging biomarker of bevacizumab efficacy in recurrent glioblastoma. Neuro Oncol (2015) 17:1139–47. doi: 10.1093/neuonc/nov028 PMC4490872 25754089

[19] Harris RJ Cloughesy TF Hardy AJ Liau LM Pope WB Nghiemphu PL. MRI Perfusion measurements calculated using advanced deconvolution techniques predict survival in recurrent glioblastoma treated with bevacizumab. J Neuro-Oncol (2015) 122:497–505. doi: 10.1007/s11060-015-1755-8 25773062

[20] Schmainda KM Zhang Z Prah M Snyder BS Gilbert MR Sorensen AG. Dynamic susceptibility contrast MRI measures of relative cerebral blood volume as a prognostic marker for overall survival in recurrent glioblastoma: results from the ACRIN 6677/RTOG 0625 multicenter trial. Neuro-Oncology (2015) 17:1148–56. doi: 10.1093/neuonc/nou364 PMC4490871 25646027

[21] Donahue KM Krouwer HG Rand SD Pathak AP Marszalkowski CS Censky SC. Utility of simultaneously acquired gradient-echo and spin-echo cerebral blood volume and morphology maps in brain tumor patients. Magnetic Resonance Med (2000) 43:845–53. doi: 10.1002/1522-2594(200006)43:6<845::AID-MRM10>3.0.CO;2-J 10861879

[22] Schmainda KM Rand SD Joseph AM Lund R Ward BD Pathak AP. Characterization of a first-pass gradient-echo spin-echo method to predict brain tumor grade and angiogenesis. Am J Neuroradiology (2004) 25:1524–32. PMC7976425 15502131

[23] Boxerman JL Schmainda KM Weisskoff RM. Relative cerebral blood volume maps corrected for contrast agent extravasation significantly correlate with glioma tumor grade, whereas uncorrected maps do not. Am J Neuroradiology (2006) 27:859–67. PMC8134002 16611779

[24] Macdonald DR Cascino TL Schold SC Cairncross JG. Response criteria for phase II studies of supratentorial malignant glioma. J Clin Oncol (1990) 8:1277–80. doi: 10.1200/JCO.1990.8.7.1277 2358840

[25] Wen PY Macdonald DR Reardon DA Cloughesy TF Sorensen AG Galanis E. Updated response assessment criteria for high-grade gliomas: Response assessment in neuro-oncology working group. J Clin Oncol (2010) 28:1963–72. doi: 10.1200/JCO.2009.26.3541 20231676

[26] Bedekar D Jensen TR Schmainda KMKM. Standardization of relative cerebral blood volume (rCBV) image maps for ease of both inter and intra-patient comparisons. Magn Reson Med (2010) 64:907–13. doi: 10.1002/mrm.22445 PMC4323176 20806381

[27] Schmainda KM Prah MA Zhang Z Snyder BS Rand SD Jensen TR. Quantitative delta T1 (dT1) as a replacement for adjudicated central reader analysis of contrast-enhancing tumor burden: A subanalysis of the American college of radiology imaging network 6677/radiation therapy oncology group 0625 multicenter brain tumor. Am J Neuroradiology (2019) 40:1132–39. doi: 10.3174/ajnr.A6110 PMC6620020 31248863

[28] Sharma A Low J Mrugala MM. Neuro-oncologists have spoken-the role of bevacizumab in the inpatient setting. A Clin economic conundrum. Neuro-Oncology Pract (2019) 6:30–6. doi: 10.1093/nop/npy011 PMC6656300 31385984

[29] Nguyen HT Nguyen N Liu LY Dovek L Lenchner D Harris R. Bevacizumab at first recurrence after standard radio-chemotherapy is associated with improved overall survival in glioblastoma patients with large tumor burden. Neuro-Oncology Pract (2019) 6:103–111. doi: 10.1093/nop/npy021 PMC6656330 31386050

[30] Liu LY Ji MS Nguyen NT Chow FE Molaie DM Pianka ST. Patterns of long-term survivorship following bevacizumab treatment for recurrent glioma: a case series. CNS Oncol (2019) 8:CNS35. doi: 10.2217/cns-2019-0007 31293169 PMC6713025

